# Interferon-α Revisited: Individualized Treatment Management Eased the Selective Pressure of Tyrosine Kinase Inhibitors on BCR-ABL1 Mutations Resulting in a Molecular Response in High-Risk CML Patients

**DOI:** 10.1371/journal.pone.0155959

**Published:** 2016-05-23

**Authors:** Vaclava Polivkova, Peter Rohon, Hana Klamova, Olga Cerna, Martina Divoka, Nikola Curik, Jan Zach, Martin Novak, Iuri Marinov, Simona Soverini, Edgar Faber, Katerina Machova Polakova

**Affiliations:** 1 Institute of Hematology and Blood Transfusion, Prague, Czech Republic; 2 Faculty of Science, Charles University, Prague, Czech Republic; 3 Department of Hemato-Oncology, Faculty of Medicine and Dentistry, Palacky University and Faculty Hospital, Olomouc, Czech Republic; 4 Institute of Clinical and Experimental Hematology of the 1st Medicine Faculty, Charles University, Prague, Czech Republic; 5 Department of Internal Medicine and Hematology, 3rd Faculty of Medicine and Faculty Hospital Kralovske Vinohrady, Charles University, Prague, Czech Republic; 6 Institute of Hematology “L. e A. Seràgnoli”, University of Bologna, Bologna, Italy; University of Thessaly, Faculty of Medicine, GREECE

## Abstract

Bone marrow transplantation or ponatinib treatment are currently recommended strategies for management of patients with chronic myeloid leukemia (CML) harboring the T315I mutation and compound or polyclonal mutations. However, in some individual cases, these treatment scenarios cannot be applied. We used an alternative treatment strategy with interferon-α (IFN-α) given solo, sequentially or together with TKI in a group of 6 cases of high risk CML patients, assuming that the TKI-independent mechanism of action may lead to mutant clone repression. IFN-α based individualized therapy decreases of T315I or compound mutations to undetectable levels as assessed by next-generation deep sequencing, which was associated with a molecular response in 4/6 patients. Based on the observed results from immune profiling, we assumed that the principal mechanism leading to the success of the treatment was the immune activation induced with dasatinib pre-treatment followed by restoration of immunological surveillance after application of IFN-α therapy. Moreover, we showed that sensitive measurement of mutated BCR-ABL1 transcript levels augments the safety of this individualized treatment strategy.

## Introduction

In 1983, Talpaz et al [1] introduced interferon-α (IFN-α) as a treatment for chronic myeloid leukemia (CML), and it became the standard CML therapy in the 90s. IFN-α modulates gene expression, promotes cell differentiation and apoptosis, inhibits cell growth and proliferation, restores regulation by the bone marrow microenvironment and induces an immunomodulatory response [2]. However, only 10–20% of treated patients achieved complete cytogenetic remission (CCgR) associated with prolonged survival [3,4]. Patients who have been successfully treated with IFN-α express increased amounts of cytotoxic T lymphocytes (CD8+ T-cells) and natural killer (NK) cells [5–7].

Targeted therapy with tyrosine kinase inhibitors (TKIs) impairing BCR-ABL1 kinase activity has greatly improved the outcome of patients with CML and has become the standard first line treatment since 2001 [8]. Approximately 20–30% of patients, however, develop resistance during TKI therapy. Approximately half of these resistant cases can be explained by point mutations in the BCR-ABL1 kinase domain [9, 10]. Notably, all currently available TKIs except ponatinib fail to overcome the most resistant mutation, T315I. Likewise, the presence of compound mutations, often resulting from sequential TKIs therapy [11, 12], can barely be overcome by TKIs, including ponatinib [13, 14].

IFN-α treatment still has its place in CML therapy in the TKI area. IFN-α is effective against dormant CML stem cells, which are resistant to TKI therapy. Essers *et al*. [15] showed that IFN-α affected dormant CML cells exit G0 phase and enter the active cell cycle with a mouse model [15]. Six clinical studies describe the use of IFN-α in combination with TKI to elicit a sustained deep molecular response and possible TKI therapy cessation [16].

The recommended treatment options for patients with the T315I mutation include ponatinib, bone marrow transplantation or clinical studies testing novel compounds [17]. However, in some individual cases, none of these approaches can be applied. We assumed that an alternative option was an individualized treatment strategy that eased the selective pressure on the mutated clone by stopping TKI and administering IFN-α alone, sequentially or together with TKI. We suggest that TKI sequential therapy with IFN-α or TKI cessation may lead to loosening of the selective pressure of TKI on the mutant clone that is resistant against TKI, but a non-mutated clone may emerge. After re-initiation of TKI, the proliferation of the non-mutated clone is inhibited, but TKI resistant mutant clones may re-emerge. Combination therapy of IFN-α and TKI yields benefits of both drugs.

In this work, we aimed to follow-up the effect of TKI cessation and solo IFN-α treatment or the combination of TKI with IFN-α on the reduction in mutant clones as assessed by using highly sensitive next-generation deep sequencing (NGS) in 6 CML cases, in which this alternative treatment strategy was applied in clinical practice. Additionally, we analyzed the immune profiles of patients at specific time points, i.e., before IFN-α initiation and during IFN-α treatment to follow-up the immunomodulatory effects of IFN-α which are supposed to contribute to the leukemic clone suppression [6, 18].

We showed that individualized IFN-α-based treatment resulted in the reduction of the T315I and M351T/F317L compound mutation burden to undetectable levels, as assessed by NGS, and in the achievement of a sustained molecular response in 4/6 high risk CML patients. Based on the observed immune-profiles, we assume that the immune changes induced by sequential treatment with the TKI (dasatinib) and IFN-α were crucial for the achievement of this favorable response. Moreover, we determined an important role for a highly sensitive measurement of BCR-ABL1 mutant mRNA load dynamics by NGS [12,19] for individualized treatment management.

## Material and Methods

### Ethics statement

This work was conducted in accordance with the principles of the Helsinki declaration and was approved by the Ethics Committees of Department of Hematology and Blood Transfusion Prague, University Hospital Olomouc and 3rd Faculty of Medicine and Faculty Hospital Kralovske Vinohrady. All patients gave their written informed consent prior to this work.NGS data are available upon request from an Ethics Board or the corresponding autor.

### Patients

Six CML patients (5 males and 1 female) (Table 1) were managed in a clinical practice based on the individual treatment scenarios of administering IFN-α after failure of 2 lines (5 patients) or 3 lines (1 patient) of TKI treatment due to the development of the highly resistant mutation T315I or compound mutations, respectively (Table 2). Patients could not switch to ponatinib due to its unavailability at that time nor could any patient be transplanted for comorbidities or lack of an HLA-compatible donor.

**Table 1:** Patient’s characteristics.

Patient no	Diagnosis	Sex	Age	Sokal score	Disease duration(months)	Response*
1	CML-AP	M	44	HR	126	MMR
2	CML-AP	F	59	HR	68	MR^5^
3	CML-CP	M	39	HR	62	MR^5^
4	CML-CP	M	23	UN	96	MMR
5	CMP-CP	M	80	IMR	44	CHR
6	CML-CP	M	65	HR	39	CHR (MMR after current ponatinib treatment)

AP—Accelerated Phase; CP—Chronic Phase; LR- low risk; IMR—intermediate risk; HR-high risk; MMR—Major Molecular Response (BCR-ABL1 ≤ 1%); MR^5^ (deep Molecular Response– 5 log reduction of BCR-ABL1 transcript levels from the standardized baseline); UN- unknown,

*–response to IFN-α individualized therapy

**Table 2:** Summary of sequential lines of treatment, therapy responses and mutation status.

Patient no.	1st line treatment		2nd line treatment		3rd line treatment		4th line treatment			5th line treatment		6th line treatment		
	*months response*	*mutation %*	*months response*	*mutation %*	*months response*	*mutation %*	*months response*	*mutation %*		*months response*	*mutation %*	*months response*	*mutation %*	
1	**imatinib**^**2**^ 13; CHR	T315I 100 E279V 2.5	**dasatinib**^**1**^ 9; MMR	T315I 100	**IFN**^**1**^ 88; MMR	UND								
2	**imatinib**^**1**^ 19;CCgR loss	M351T 99 S500F 3.0 I360V 7.0	**dasatinib**^**1**^ 22; CCgR loss	M351T/F317L 100	**nilotinib**^**4**^ 3; CCgR	M351T/ F317L 100	**IFN**^**2**^**/nilotinib**^**4**^ 14; MR^5^	UND	**IFN**^**2**^**/nilotinib**^**3**^15; MR^5^		UND			
3	**imatinib**^**2**^ 36; CCgR loss	M244V 11 G250E 4.0 T315I 15	**dasatinib**^**1**^ 6; CHR	T315I 98	**IFN**^**1**^ 7; CCgR	T315I 48 G250E 1.0	**IFN**^**1**^**/nilotinib**^**1**^ 23; MR^5^	UND						
4	**imatinib**^**1**^ 13; CHR	L248V 35 T315I 5.6	**dasatinib**^**1**^ 15; CHR	T315I 100	**IFN**^**1**^ 14; CHR	UND	**IFN**^**3**^**/nilotinib**^**4**^ 8; CHR	UND	**nilotinib**^**2**^ 46; MMR		UND			
5	**imatinib**^**1**^ 18; CCgR loss	T315I 98 E255V 8.6 H246Y 4.0 S438F 3.0	**nilotinib**^**4**^ 1; CHR	T315I 99 E255V 1.0	**IFN**^**1**^**/nilotinib**^**4**^2; CHR	T315I 98 E255V 2.0	**IFN**^**1**^ 18; CHR	T315I 4.0 E255V 17 F317L 60	**imatinib**^**1**^ 3; CHRᵻ		E255V 9.2 F317L 80			
6	**imatinib**^**1**^ 1; CHR	NA	**nilotinib**^**2**^ 10; MMR loss	T315I 20 Y253H 28 E255V 18 F359I 25	**IFN**^**2**^ 6; CCgR loss	T315I 96 F359I 3.0	**IFN**^**1**^**/dasatinib**^**2**^ 3; CHR	T315I 100	**IFN**^**1**^**/HU** 6; CHR		T315I 100	**ponatinib**7;CCgR		T315I 100

CHR—complete hematologic response; CCgR—complete cytogenetic response; MMR- major molecular response; MR5 –deep molecular response, i.e. 5 log reduction of BCR-ABL1IS transcript levels from the baseline; imatinib^1^—400mg/day; imatinib^2^- 600mg/ day; dasatinib^1^—100mg/day; dasatinib^2^—60mg/day; IFN^1^ –interferon-α: 3MU/ day; IFN^2^ -interferon-α: 3x 3MU/week; IFN^3^ –interferon-α: 4,5MU/day; HU- hydroxyurea; nilotinib^1^-300mg/day; nilotinib^2^—400mg/day; nilotinib^3^—600mg/day; nilotinib^4^—800mg/day; ponatinib-30mg/day; NA- sample was not available for the analysis; UND- undetectable levels of mutated BCR-ABL transcripts by NGS; ᵻ exitus

### Standardized measurement of BCR-ABL1 transcript levels

BCR-ABL1 transcript levels were measured regularly every 3 months during the treatment [20]. Total RNA was prepared from the total leukocytes of peripheral blood by Trizol (Thermofisher scientific, Waltham, MA, USA) lysis-phenol-chloroform extraction; cDNA was synthesized by M-MLV reverse transcriptase (Promega Corporation, Madison, WI, USA) using random hexamer primers (Jena Bioscience, Jena, Germany). GUSB (in Prague lab) or ABL1 (in Olomouc lab) were used as the control genes for the standardized quantification of BCR-ABL1 mRNA using reverse-transcriptase qPCR. BCR-ABL1 quantification was standardized in both labs within the EUTOS for the CML project of ELN, and data are reported in the International Scale (IS) [21]. Primers and probes for BCR-ABL1, GUSB and ABL1 were applied according to the Europe Against Cancer recommendations and commercial plasmid standards were used to perform calibration curves (Ipsogen, Qiagen, Valencia, CA, USA).

### BCR-ABL1 mutation detection by next-generation deep sequencing

The dynamics of the mutation load were analyzed both retrospectively and prospectively using NGS (GS Junior, Roche Applied Science, Basel, Switzerland) of the cDNA region encoding the kinase domain of BCR-ABL1 in a total of 54 samples (median, 6 samples per patient; range, 5–15) of peripheral blood leukocytes according to a previously established protocol [12,19]. Briefly, the mRNA was transcribed using random hexamers and SuperScript II enzyme (Invitrogen, Thermofisher scientific,Waltham, MA, USA). In order to analyze the KD of only the translocated ABL1, a nested PCR approach was used. The first selective amplification was performed with a forward primer located on the BCR exon 13 (5’-TGACCAACTCGTGTGTGAAACTC-3’) and a reverse primer located on the ABL1 exon 11 (5’-ATCTCAGGCACGTCAGTGGT-3’). The second amplification step was subsequently performed using 4-amplicon assays developed and distributed within the framework of the IRON-II phase study (Interlaboratory RObustness of Next-Generation Sequencing) [22]. The required minimal level of total BCR-ABL1 transcripts in the analyzed sample was 0.1%^IS^.

NGS data were evaluated using Amplicon Variant Analyzer software (Roche Applied Science) and also using pipeline that was described in the recent paper [19]. For error filtering (correction for false positive variants) was used algorithm [19] that takes into account distribution of errors in sequencing data from healthy donor samples in kinase domain of ABL1. Negative binomial distribution fit to those data was used for error filtering.

### Flow cytometry

The basic immune-profiling was performed in 5/6 CML patients using flow-cytometry that detected populations of CD4+, CD8+, regulatory T-cells (Tregs) and NK cells before IFN-α initiation and during IFN-α treatment. Peripheral blood samples were stained with anti-CD3 FITC clone MEM-57, anti-CD4 FITC clone MEM-241, anti-CD8 PE clone MEM-31, anti-CD16+CD56+ PE clones LNK-16 and MEM-188, anti-CD4 APC clone MEM-24 and anti-CD8 PerCP clone MEM-31 (Exbio Prague, Czech Republic) using a lyse/no-wash protocol. Minimal acquisition was designated as 10,000 CD3+ events. Intracellular Foxp3 analyses were performed using a Human Regulatory T Cell Staining Kit (eBioscience, San Diego, CA, USA) which contains the Foxp3 PE clone PCH101, CD4 FITC and CD25 APC antibodies plus buffers and controls. The staining protocol was optimized with PBMCs. Fresh Ficoll prepped PBMCs were surface stained with anti- CD25 APC and anti-CD4FITC antibodies and then subsequently with anti Foxp3 PE antibody (intracellular staining). CD25+CD4+Foxp3+ cells were considered as T regulatory cells. Minimal acquisition was designated as 10,000 CD25+ CD4+ events. Analysis of immune competent cell subpopulations was performed using the FACS Diva (BD Biosciences, San Jose, CA, USA) software. Absolute numbers were expressed as the number of cells ×10^9^/L.

## Results

In this group of 6 cases, 5 patients failed therapy due to the acquisition of a T315I mutation, and one patient failed therapy due to the acquisition of a compound mutation M351T/F317L during sequential TKI treatment. None of the 6 patients could be switched to ponatinib due to its unavailability at that time, nor could any patient be transplanted for comorbidities or lack of an HLA-compatible donor.

**Fig 1:**
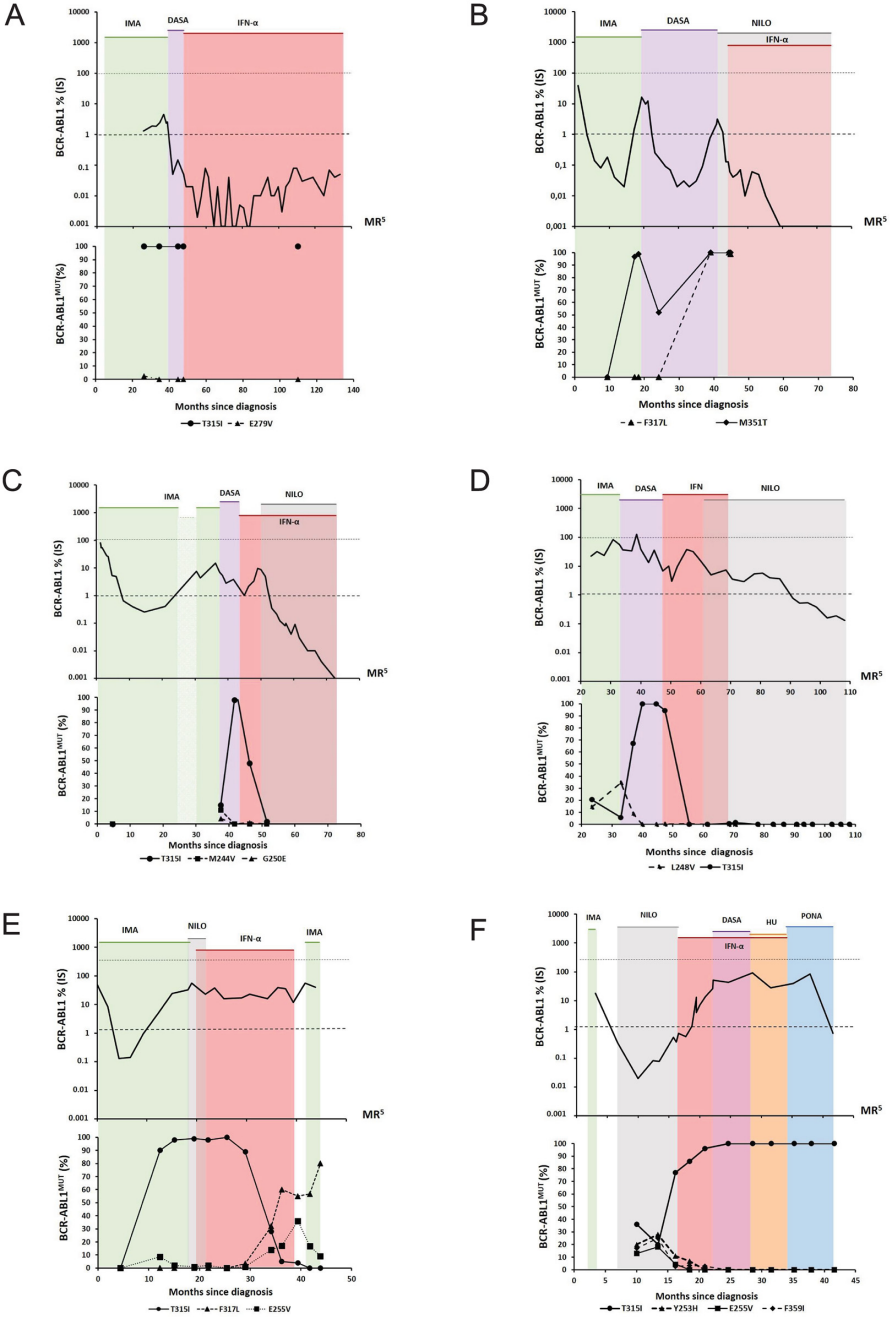
Dynamics of total and mutated BCR-ABL1 transcript levels. (A) Patient no 1: The patient achieved MMR on solo IFN-α and maintained MMR for 88 months; the T315I mutation was persistently undetectable for 62 months due to the overall low levels of total BCR-ABL1 transcripts. (B) Patient no 2: Compound mutations M351T/F317L (100%) developed after sequential therapy with imatinib and dasatinib. The patient has now been on IFN-α/nilotinib therapy with undetectable mutations for 29 months. MR^5^ was achieved on the 15^th^ month of the combined treatment. (C) Patient no 3: Poor compliance to imatinib treatment within the 24^th^– 30^th^ month; the T315I mutation burden decreased on solo IFN-α therapy down to undetectable levels after the combination of IFN-α and nilotinib. The patient achieved MR^5^ in the 22^nd^ month from IFN-α treatment initiation. (D) Patient no 4: The T315I mutation decreased on solo IFN-α therapy and was not detected for the subsequent 46 months on solo nilotinib therapy. The relatively slow reduction of the BCR-ABL1 transcript level and MMR achievement might have been caused by a problematic compliance to nilotinib. (E) Patient no 5: The T315I mutation decreased on solo IFN-α therapy, but the F317L and E255V mutations appeared and expanded. Death of this patient was related to lung tuberculosis. (F) Patient no 6: IFN-α therapy did not contribute to the T315I reduction and response improvement. Therefore, the patient has been switched to ponatinib with CCgR achievement after 7 months from ponatinib treatment initiation. IMA-imatinib, NILO-nilotinib, DASA-dasatinib, IFN-α –interferon alpha, HU-hydroxyurea, PONA-ponatinib. Note: A mutation burden of 0% represented undetectable levels of mutated BCR-ABL1 transcripts when the sequencing depth was 1,000 to 8,000 sequence reads per each nucleotide position.

TKI stoppage and introduction of an IFN-α based treatment strategy led to a major molecular response (MMR) or deep MR [23] as well as to undetectable levels of BCR-ABL1^T315I^ and BCR-ABL1^M351T/F317L^ in 4 patients (patients no 1–4, Table 2 and Fig 1).

In patient no 1 (Fig 1A), 100% of BCR-ABL^T315I^ was retrospectively detected during the first line of imatinib treatment (at the time the patient failed imatinib, BCR-ABL1 KD mutation screening using standardized Sanger sequencing was not yet available as a routine diagnostic). Surprisingly, the patient achieved MMR after the switch to dasatinib, although the total BCR-ABL1 transcripts were mutated. Because of the molecular progression with the loss of MMR and the confirmed presence of the T315I during dasatinib treatment, the patient was switched to IFN-α monotherapy which led to deep MR. During a period of evident BCR-ABL1 transcript level oscillations (from negative to positive levels up to maximum 0.04%^IS^) (Fig 1), decreased levels of CD3+ T-lymphocytes and elevated levels of NK cells were observed (Table 3). These results are comparable with long-term treatment with IFN-α [6]. Although we do not have data regarding immune profiling during dasatinib treatment, we assume that dasatinib activated immune surveillance, which may explain the MMR achievement despite the presence of T315I leukemic clones.

**Table 3:** Flow cytometry results.

Patient no	Month after diagnosis	Therapy	CD3+ T- lymphocytes 10^9/l	CD4+ T- helper cells 10^9/l	CD8+ T- cytotoxic cells 10^9/l	CD56+,CD16+NK cells 10^9/l
1	86	IFN	0,787	0,419	0,419	0,140
	119	IFN	0,783	0,537	0,290	0,131
	122	IFN	1,06	0,532	0,616	0,227
2	20	DASA	0,912	0,572	0,465	0,465
	26	DASA	1,164	0,543	0,718	0,66
	28	DASA	1,746	0,95	0,95	0,616
	32	DASA	1,246	0,659	0,712	0,338
	65	IFN+NILO	1,123	0,343	0,439	0,137
3	38	DASA	0,465	0,268	0,255	0,469
	39	DASA	0,808	0,413	0,430	0,705
	43	IFN	0,841	0,390	0,369	1,190
	57	IFN+NILO	0,632	0,303	0,320	0,142
4	93	NILO	1,06	0,52	0,48	0,16
	96	NILO	1,25	0,66	0,54	0,14
5	42	IMA	1,07	0,72	0,32	0,06

Note: The flow cytometry analysis of patient no 6 could not be performed due to unavailability of samples. IMA-imatinib, NILO-nilotinib, DASA-dasatinib, IFN -interferon alpha.

The combination of IFN-α/nilotinib was applied for patient no 2 (Fig 1) after failure of imatinib first line and dasatinib second line therapies. IFN-α/nilotinib resulted in the achievement of MR^5^, thus, the BCR-ABL1 mutation could not be detected. Elevated levels of NK and CD8+T cells (Table 3) were detected in this patient, probably representing an immune activation by dasatinib treatment followed by restoration of immunological surveillance by combined IFN-α/TKI treatment.

Solo IFN-α was administered in patient no 3 (Fig 1) after second line dasatinib failure. The levels of BCR-ABL1^T315I^ decreased, while the total non-mutated BCR-ABL1 transcript levels increased on solo IFN-α. For this reason, the therapy was changed to the combination of IFN-α/nilotinib and the patient achieved MR^5^. This, as well as a previous case, outlined a possible mechanism of action for both drugs when TKI and IFN-α were co-administered; TKI controls un-mutated BCR-ABL1 positive clones and IFN-α controls mutated BCR-ABL1 clones exhibiting resistance to TKI. The immunological analysis of patient no 3 could be performed both during dasatinib therapy and during co-administration of IFN-α/nilotinib. The relative and absolute representation of CD3+ T-cells was reduced with gradual normalization (Table 3). The ratio of CD4+/CD8+ T-lymphocytes was constantly reduced and representations of both populations were generally similar. Importantly, the number of NK cells was markedly accentuated during dasatinib treatment.

IFN-α solo therapy resulted in a decrease of the T315I mutation to undetectable levels in patient no 4 (Fig 1). Its co-administration with nilotinib was later applied in consideration of persistently high total BCR-ABL1 mRNA levels. IFN-α was stopped after 8 months due to undetectable mutations as shown by NGS. The patient has been on solo nilotinib therapy for 46 months, with undetectable T315I (or other) mutations and MMR achievement. Immune profiling showed slightly accentuated levels of NK cells (Table 3). In this case, we can only speculate about the involvement of a particular cell fraction to control CML clones.

During IFN-α monotherapy, the T315I mutation levels were reduced from 100% to 4% in patient no 5 (Fig 1), but two other mutations appeared (F317L and E255V). This 85-year old patient died of lung tuberculosis with an overall survival of 44 months. We observed a basic division of T-lymphocyte fractions during treatment management. The best IFN-α therapy outcome was described among others in patients younger than 60 years [24]. The enhancing effect of IFN-α on the immune response was probably deficient in this elderly patient. In addition, the higher incidence of mutations may be related to advanced age.

The concept of TKI cessation and IFN-α introduction did not contribute to the T315I reduction or to response improvement in patient no 6 (Fig 1). Recently, it was possible to switch therapy to ponatinib with a reduction of total BCR-ABL1 transcripts to MMR levels, which was associated with a simultaneous reduction of T315I.

## Discussion

IFN-α CML therapy currently has an important place in CML treatment for its unique activity against dormant CML stem cells and for its ability to activate the specific immunity that is necessary for a sustained deep molecular response and possible TKI therapy cessation, as is currently being investigated in clinical trials [2].

In this work, using a series of case reports, we described that stopping TKI treatment and administering IFN-α alone, sequentially or together with TKI, may lead to reduced selective pressure by TKI on the mutant clone that is highly resistant against TKIs and that the immunomodulatory effect of IFN-α may suppress leukemic clones, which may contribute to the molecular response achievement.

The switch of TKI to IFN-α-based treatment resulted in decreased mutated BCR-ABL1 mRNAs to undetectable levels in 4/6 patients. Three of those patients achieved a major molecular response with undetectable BCR-ABL1 mutations. Based on the detection of non-mutated BCR-ABL1 transcripts, IFN-α remained withdrawn with solo nilotinib in 1/4 patient who also achieved MMR. One case of an MMR responding patient on solo IFN-α treatment showed decreased levels of CD3+ T-lymphocytes and elevated levels of NK cells. Another two patients who achieved MMR after IFN-α showed elevated levels of NK cells, likely representing immune activation on dasatinib pre-treatment followed by restored immunological surveillance after the application of combined treatment IFN-α with TKI. In 2/6 cases, TKI cessation and IFN-α introduction did not contribute to the reduction in BCR-ABL1 mutations or an improved response.

Expansion of CD8+ T-cells or NK cells was found in a proportion of dasatinib treated patients and was associated with a superior therapy response [25]. Dasatinib may induce a reversible state of aberrant immune reactivity by inhibiting immune-regulatory kinases. This activity is associated with good clinical responses and a distinct adverse effect profile [26]. Kreutzman et al. [6] reported an increased amount of CD8+ T-cells associated with good therapeutic outcomes in IFN-α treated patients. A good therapeutic effect of IFN-α therapy is associated with an increased amount of NK cells, specifically clonal γδ+ T-cells [6]. We suppose that the molecular response of patients no 1–3 was induced by the immune activation by dasatinib pre-treatment followed by restoration of immune surveillance after application of IFN-α-based therapy.

Only 2 case reports on the use of IFN-α in CML treatment in patients with the T315I mutation have been published [18,27]. Here, we report an undisputed positive effect of individualized IFN-α therapy given alone, in combination with TKI or sequentially, on the elimination of highly resistant mutational clones and in the achievement of a molecular response in 4 cases of high risk CML patients. The immune response supports the suggestion that IFN-α therapy after dasatinib pretreatment can help restore activated immune surveillance. In addition to reducing the TKI selective pressure on mutant clones, this strategy contributed to the achievement of a molecular response. We believe that the individualized treatment management described herein using IFN-α as a monotherapy or in combination with a TKI represents an important alternative treatment strategy in some cases of high risk CML patients that may have limited options, notably, the inability to receive ponatinib or a bone marrow transplantation. We also show that the best patient management results from an integrated molecular approach combining highly sensitive NGS-based analysis of mutation dynamics and standardized RTq-PCR for molecular response monitoring.
